# Interplay between Influenza Virus and the Host RNA Polymerase II Transcriptional Machinery

**DOI:** 10.1016/j.tim.2018.12.013

**Published:** 2019-05

**Authors:** Alexander P. Walker, Ervin Fodor

**Affiliations:** 1 Sir William Dunn School of Pathology, University of Oxford, South Parks Road, Oxford OX1 3RE, UK

**Keywords:** influenza virus, RNA polymerase, cap snatching, Pol II, CTD

## Abstract

The influenza virus RNA-dependent RNA polymerase (RdRP) cleaves the 5′ end of nascent capped host RNAs and uses the capped RNA fragment to prime viral transcription in a mechanism called ‘cap snatching’. Cap snatching requires an intimate association between influenza RdRP and cellular RNA polymerase II (Pol II), which is the source of nascent capped host RNAs targeted by influenza virus. Recent structural studies have revealed how influenza RdRP binds to Pol II and how this binding promotes the initiation of viral transcription by influenza RdRP. In this review we focus on these recent insights into the mechanism of cap snatching by influenza virus and the impact of cap snatching on host gene expression during influenza virus infection.

## Highlights

Influenza virus RdRP associates with cellular Pol II. This provides influenza RdRP with a source of nascent capped host RNAs which it targets in a process called ‘cap snatching’ to generate primers for viral transcription.

Influenza RdRP makes direct interactions with the Pol II C terminal domain (CTD). Molecular details of these interactions have recently been determined, and there is evidence that Pol II CTD binding promotes viral transcription by modulating the conformation of influenza RdRP.

Influenza RdRP cap snatches small nuclear/nucleolar RNAs most frequently. When cap snatching generates suboptimal capped RNA fragments, influenza RdRP can use a prime-and-realign mechanism to promote transcription initiation.

Influenza virus infection reduces Pol II abundance in gene bodies, suggesting that cap snatching contributes to host shut-off.

## Influenza Virus Transcription

Influenza viruses are negative-strand RNA viruses of the family Orthomyxoviridae. Influenza A and B viruses are responsible for annual epidemics which cause up to half a million deaths worldwide each year. Influenza C viruses are less prevalent and they usually cause mild infections, while influenza D viruses are not known to infect humans [1]. Vaccines against influenza viruses causing annual epidemics are available, though variation in the haemagglutinin and neuraminidase surface proteins, the major antigenic determinants of influenza viruses, can render vaccines ineffective 2, 3.

Influenza viruses have a genome consisting of eight viral RNA (vRNA) segments, each encoding one or two major viral proteins. Each genome segment is packaged with viral nucleoprotein (NP) and the RdRP in viral ribonucleoprotein complexes (vRNPs). Influenza virions must contain a full complement of eight vRNPs to be productively infectious. Virions bind to sialic acid on the cell surface, are endocytosed, and fuse with the endosomal membrane. vRNPs are released into the cytoplasm and are trafficked to the nucleus where they begin synthesising viral mRNAs to produce viral proteins [4]. Once viral proteins have accumulated in the later stages of infection, viral mRNA production decreases and replication of the viral genome segments increases.

Influenza RdRP is a heterotrimer consisting of a core polymerase subunit with a canonical right-hand fold (PB1), and two auxiliary subunits, PB2 and PA [4]. The RdRP is responsible for replicating the negative-sense vRNA genome, a two-step process that requires RdRP oligomerisation and involves the synthesis of a positive-sense complementary RNA (cRNA) 5, 6, 7. The RdRP also transcribes each of the vRNA genome segments, producing positive-sense viral mRNAs with a 5′ terminal N7-methyl guanosine (m^7^G) cap and 3′ polyA tail 4, 8. As a result of these modifications, viral mRNAs are structurally identical to host mRNAs and are therefore able to exploit endogenous cellular pathways for processing and nuclear export 9, 10. The 3′ polyA tail is added by the RdRP when it stutters on a short uridine tract present near the 5′ terminus of all viral genome segments. Stuttering results in polyadenylation of the viral mRNA and transcription termination 11, 12.

For 5′ m^7^G capping of mRNAs, some negative-sense single-stranded RNA (ssRNA) viruses, such as Ebola, measles, and rabies viruses, synthesize an m^7^G cap *de novo* using their own viral mRNA capping machinery [13]. However, influenza viruses do not have their own capping enzymes; instead, they ‘snatch’ the m^7^G capped 5′ end of nascent host RNAs 14, 15. Influenza RdRP binds to m^7^G on nascent host RNAs using a cap-binding domain on the RdRP PB2 subunit 16, 17, 18, 19. It then cleaves the host RNA 10–13 nt downstream of the 5′ cap using an endonuclease domain at the N terminus of the RdRP PA subunit 20, 21, 22. The resulting short 5′ capped RNA fragment is used as a primer to initiate transcription of the viral genome segment by influenza RdRP [15]. This mechanism is called ‘cap snatching’.

Cap snatching by the influenza RdRP requires an intimate association with the host transcriptional machinery 23, 24. Host RNA polymerase II (Pol II) synthesizes RNAs which are 5′ m^7^G capped and 3′ polyadenylated, and nascent Pol II transcripts with a completed m^7^G cap are the target of cap snatching by influenza RdRP. Understanding of influenza RdRP interactions with Pol II at a molecular level has improved greatly in recent years, thanks to biochemical studies and multiple crystal structures of the complete influenza RdRP heterotrimer 25, 26, 27. Here we review novel insights into the association of influenza RdRP with Pol II, mechanisms of viral transcription initiation, and impacts of influenza virus infection and cap snatching on host gene expression.

## Targeting Host Transcriptional Machinery

Cellular Pol II is a complex composed of 12 core subunits. The **RPB1** subunit (see Glossary) has a flexible C terminal domain (CTD) consisting of 52 heptad repeats in humans. This CTD, with the consensus Tyr1–Ser2–Pro3–Thr4–Ser5–Pro6–Ser7 for the heptad repeats, acts as a landing pad for cellular transcription cofactors. Among others, the CTD can be phosphorylated at Ser2 and Ser5 residues. Different phosphorylation states mark the stage of transcription, so specific sets of cellular proteins can be recruited as required [28].

Early in Pol II transcription, **CDK7** (a component of **TFIIH**) phosphorylates Ser5 (Ser5P) on the Pol II CTD. The Ser5P CTD modification is recognised by cellular machinery required early in Pol II transcription, such as mRNA capping enzymes [29]. Pol II can pause transcription 30–60 nt downstream of the transcription start site, which is referred to as promoter-proximal pausing. The **P-TEFb** complex releases paused Pol II so it can continue transcription [30]. **CDK9**, a component of the P-TEFb complex, phosphorylates Ser2 on the Pol II CTD (Ser2P). The Ser2P CTD modification occurs on elongating Pol II, and Ser5 on the CTD is gradually dephosphorylated throughout elongation 28, 31.

**Figure 1:**
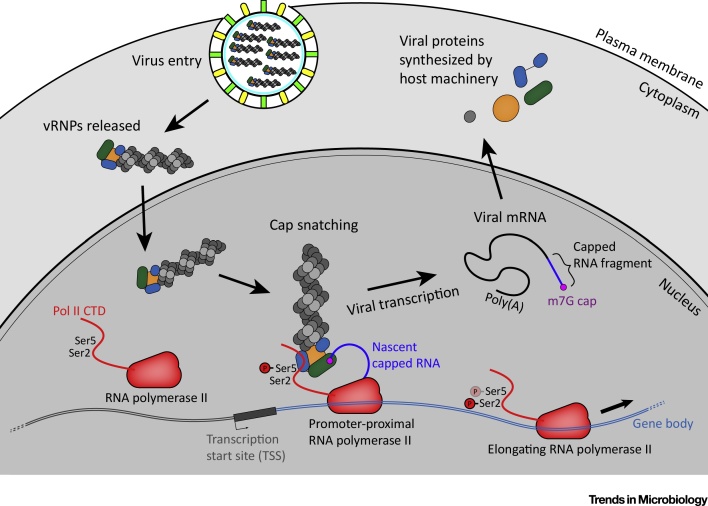
The Association of Influenza vRNPs with Cellular Pol II. Influenza virions bind to receptors at the plasma membrane and are endocytosed. Viral and endosomal membranes fuse, releasing vRNPs into the cytoplasm. vRNPs are then trafficked to the nucleus to target cellular Pol II. The CTD of unengaged Pol II is hypophosphorylated at Ser2 and Ser5. Pol II initiates at the transcription start site, and is Ser5-phosphorylated early in transcription. Later in transcription elongation, Ser2 is phosphorylated and Ser5 is gradually dephosphorylated. Influenza vRNPs in the nucleus target the Ser5-phosphorylated Pol II CTD, and bind to the m^7^G cap of nascent RNA on Pol II. RdRP in the vRNP cap snatches the nascent RNA, which is then used to prime viral transcription by influenza RdRP. The m^7^G capped, polyadenylated viral mRNA is exported to the cytoplasm through host pathways, and translated by host machinery.

The Ser5P CTD modification on Pol II is most prevalent close to transcription start sites, but is still present at lower abundance in gene bodies [31]. Influenza RdRP is able to interact directly with Pol II by binding to the CTD, specifically when the CTD has the Ser5P modification. This interaction was first described using the RdRP only, and has since been demonstrated with RdRP in the context of vRNPs using pulldown and coimmunoprecipitation assays 24, 25, 32. It is thought that this interaction is involved in localising vRNPs to Pol II, a source of nascent host RNAs, for cap snatching (Figure 1).

The specificity of influenza RdRP for Ser5P CTD indicates that cap snatching most likely occurs early in Pol II transcription. In agreement with this, chromatin immunoprecipitation (ChIP) data show that influenza RdRP is primarily associated with regions close to gene promoters [33]. Currently, interactions between influenza RdRP and Pol II are only known to be mediated by the Pol II CTD. However, it is likely that vRNPs and Pol II make further interactions, possibly mediated by other cellular proteins. Influenza RdRP interaction studies have identified several Pol II-associated factors, including CDK9, **HTat-SF1** and **SPT5** 34, 35, 36. SPT5 is involved in recruiting the mRNA capping complex, and CDK9 is a component of the P-TEFb complex. Influenza RdRP also interacts with the **RNA exosome complex**, involved in RNA quality control in the nucleus, which may enhance the targeting of influenza RdRP to gene promoters for subsequent cap snatching [37].

## Molecular Mechanisms of Cap Snatching and Viral Transcription Initiation

Improved understanding of influenza RdRP structure has led to elucidation of the molecular mechanisms of cap snatching. Recent structural studies have revealed how the RdRPs of different influenza types bind to the Pol II CTD, and how this interaction promotes cap snatching and viral transcription initiation at a molecular level.

### How Does the Pol II CTD Associate with Influenza RdRP?

**Figure 2:**
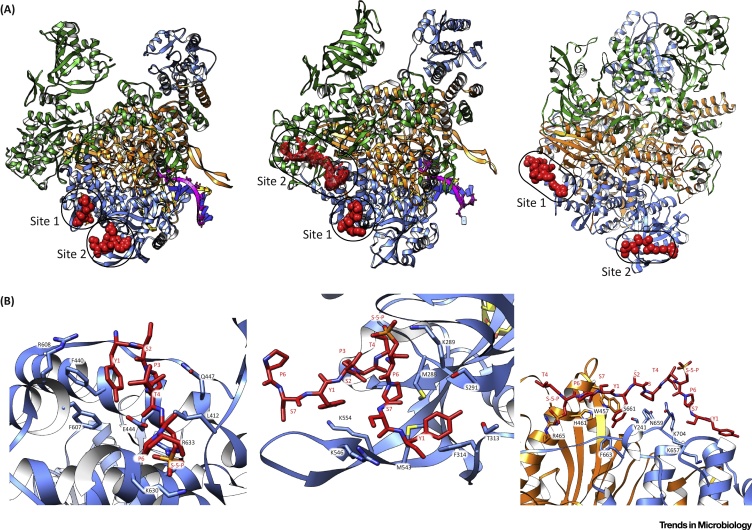
Structures of Influenza RdRP Pol II CTD Binding Sites. (A) Ribbon structures of FluA RdRP (left) (PDB:5M3H), FluB RdRP (centre) (PDB:5M3J), and FluC RdRP (right) (PDB:6F5P) bound to Pol II CTD peptides. The PB1 subunit is coloured orange, PB2 subunit green, and PA subunit blue. CTD peptides are shown as red spheres, or mesh for the unresolved density on FluB RdRP. (B) Detailed views of CTD Site 1 (left) and Site 2 (centre) on FluA RdRP, and Site 1 on FluC RdRP (right).

The interaction between influenza RdRP and Pol II CTD was shown to be direct by using Pol II CTD mimic peptides [25]. Cocrystallisation of influenza RdRP with Pol II CTD mimic peptides has resulted in a first glimpse of the structural basis of this interaction. The structure of bat influenza A (FluA) RdRP complexed with a Ser5P CTD mimetic revealed two binding sites, both on the RdRP PA subunit (Figure 2A, left). Site 1 is a pocket formed by several α-helices on the PA subunit (Figure 2B, left), while Site 2 is more distant from the PB1 core, located behind a β-hairpin loop (Figure 2B, centre). Both sites have basic residues making interactions with the phosphate modification on Ser5 of the CTD peptide, providing the structural basis for Ser5P specificity. Mutating residues in these sites causes a loss of viral mRNA transcription by influenza RdRP, confirming that the identified Pol II CTD binding sites have a function in viral transcription. The authors suggest that a single CTD peptide could occupy both binding sites, with the central region of the CTD peptide unresolved in the structure [26]. Indeed, when influenza RdRP binds to the CTD of Pol II during cap snatching, both sites are most likely to be bound by the same Pol II CTD. The conformation of the Pol II CTD outside of these two binding sites is currently unknown.

Critical amino acid residues identified in the CTD binding sites are conserved across most FluA strains. Interestingly, amino acid sequence alignments predict that only Site 1 is conserved in influenza B (FluB) RdRP [26]. FluB RdRP was cocrystallised with the same Pol II CTD peptide, which revealed that a site similar to FluA RdRP Site 1 was indeed present, while no CTD peptide was present in the site corresponding with FluA RdRP Site 2. However, the CTD peptide-bound FluB RdRP showed an additional region of density which does not match either of the known CTD binding sites on FluA RdRP. This density appears to lie across the interface between the PB2 subunit and the C terminal region of the PA subunit, though low resolution means that the CTD peptide conformation is unresolved. This region has been referred to as Site 2 for FluB RdRP (Figure 2A, centre) [26].

Amino acid sequence alignment of the FluA RdRP CTD binding sites with influenza C (FluC) RdRP indicates that neither Site 1 nor Site 2 is strongly conserved in the FluC RdRP 26, 27. However, biochemical studies show that FluC RdRP binds Ser5P Pol II CTD peptides directly, similarly to FluA RdRP [25], raising the question of where the Pol II CTD binding sites are on FluC RdRP.

Recently, FluC RdRP has also been cocrystallised with a Pol II CTD peptide [27]. This structure confirms that FluC RdRP has Ser5P CTD binding sites distinct from those on FluA or FluB RdRPs (Figure 2A, right). Site 1 on FluC RdRP is primarily located at the interface between the PB1 and P3 (equivalent to PA in FluA) subunits (Figure 2B, right). This site is located close to the unresolved Site 2 on FluB RdRP, though it appears structurally distinct. In addition, amino acid sequence alignments show that key residues in FluC Site 1 are not conserved in FluB RdRP. A second CTD binding site is visible in the FluC RdRP–CTD complex, named Site 2, which is in a similar position to Site 2 on FluA RdRP. Side chains of the CTD peptide in Site 2 are not modelled in the FluC RdRP–CTD structure, though amino acid sequence alignments show no conservation of the FluA RdRP CTD sites in FluC RdRP, suggesting a different mode of binding [27].

These studies show that the mode of influenza RdRP binding Pol II CTD has diverged between influenza virus types. Despite this, the RdRPs of influenza A, B, and C appear to have two Pol II CTD binding sites. Serna Martin *et al*. suggest that the presence of multiple binding sites may be a mechanism to increase avidity of the Pol II CTD and influenza RdRP interaction [27].

### Conformational Regulation of RdRP by Pol II CTD

Recent evidence shows that influenza RdRP binding to Ser5P Pol II CTD promotes cap snatching and transcription initiation activity *in vitro*. These data led to the suggestion that binding to the Pol II CTD may influence the conformation of influenza RdRP in order to improve the efficiency of cap snatching and viral transcription initiation [27].

**Figure 3:**
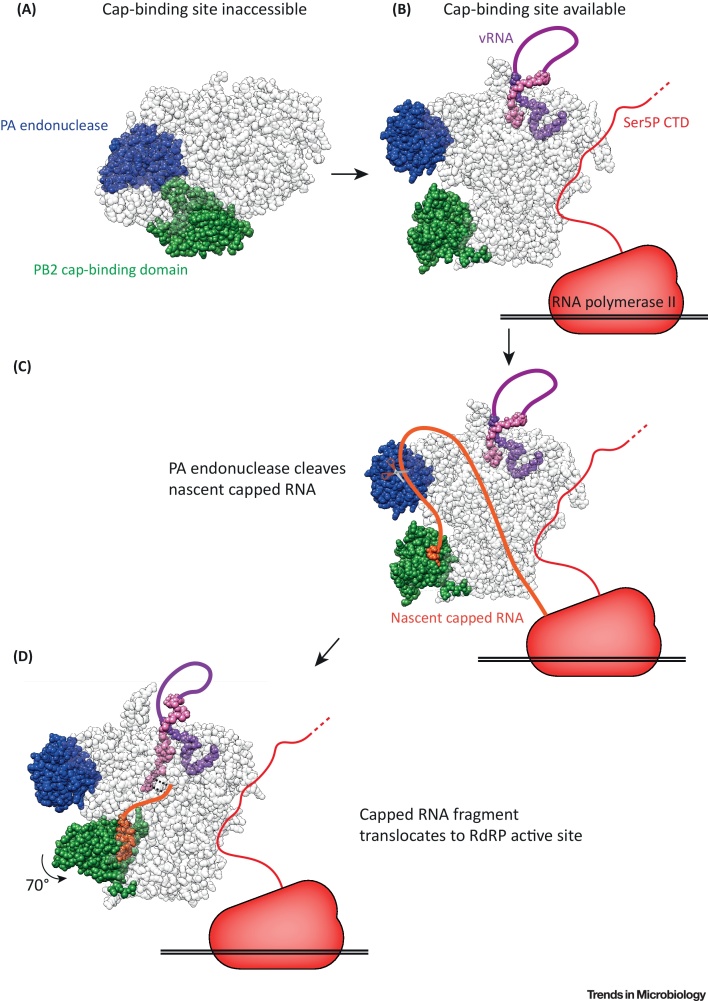
Influenza RdRP Conformational Rearrangements during Cap Snatching. (A) In the transcriptionally inactive RdRP conformation, the PB2 cap-binding domain (green) is inaccessible and cannot bind capped RNA (PDB:5D98). (B) When influenza RdRP binds to vRNA and Ser5P Pol II CTD the transcriptionally active conformation is favoured. In this conformation the PB2 cap-binding domain is accessible, and is orientated towards the PA subunit endonuclease domain (blue) (PDB:4WSB). (C) Influenza RdRP binds to nascent capped RNA (orange), then the PA endonuclease cleaves 10–13 nt downstream of the m^7^G cap (PDB:6EVK). (D) Following cleavage, the PB2 cap-binding domain rotates by 70 degrees. This moves the capped RNA fragment away from the PA endonuclease and into the RdRP active site through the product exit channel. The capped RNA fragment can then base pair with the 3′ end of the vRNA template (pink) in the active site (PDB:5MSG).

Influenza RdRP can adopt two radically different conformations, designated as ‘transcriptionally inactive’ and ‘transcription preinitiation’ conformations 38, 39, 40. The RdRP PB2 subunit, which has an m^7^G cap-binding domain, and the N terminal PA endonuclease undergo major rearrangements between the two conformations. In the ‘transcriptionally inactive’ conformation, the PB2 cap-binding domain is occluded by an adjacent linker region of the PB2 subunit 39, 40. The ‘transcription preinitiation’ RdRP conformation is compatible with cap snatching, as the cap-binding domain is accessible for binding capped host RNAs, and the PA endonuclease is correctly oriented to carry out endonucleolytic cleavage [38]. Serna Martin *et al*. propose that Ser5P Pol II CTD binding, in combination with vRNA promoter binding, promotes a ‘transcription preinitiation’ RdRP conformation to facilitate cap snatching when close to a source of nascent capped host RNAs (Figure 3) [27].

This phenomenon has been demonstrated using FluA and FluC RdRPs. In Pol II CTD Site 1 on FluC RdRP, the CTD peptide makes contacts with both P3 and PB1 subunits. The authors suggest that the CTD peptide in FluC RdRP Site 1 can also make contacts with the PB2 subunit, exclusively when FluC RdRP is in a ‘transcription preinitiation’ conformation, presenting a model for how the CTD peptide could selectively stabilise the ‘transcription preinitiation’ conformation [27]. However, it is unclear whether CTD peptide in the FluA RdRP binding sites is able to make comparable interactions, so the mechanism for conformational regulation by Pol II CTD in FluA RdRP is unknown.

### Linking Cap Snatching to Transcription Initiation

Once a nascent host capped RNA is bound by influenza RdRP it is cleaved by the endonuclease domain at the N terminus of the PA subunit. The 3′ end of the resulting capped RNA fragment is used to prime viral transcription. For this to occur, the 3′ end of the capped RNA fragment must translocate into the RdRP active site, located in the core of the PB1 subunit. To achieve this, the cap-binding domain is thought to rotate by 70 degrees, as observed in multiple RdRP crystal structures 41, 42. This rotation changes the position of the capped RNA fragment such that the 3′ end points towards the RdRP active site, entering through the product exit channel [43]. This also moves the capped RNA fragment away from the PA endonuclease domain, preventing further truncation of the capped RNA (Figure 3).

To prime viral transcription, the 3′ end of the capped RNA fragment must base pair with the 3′ end of the vRNA template. One or two base pairs form in the active site, such that transcription initiates opposite C2 or G3 on the 3′ end of the vRNA template. The efficiency of this base pairing has been proposed to be a factor in selecting which capped RNA fragments are successful in initiating viral transcription 44, 45, 46. Currently, RNA in the active site of the RdRP-capped RNA fragment complex is not resolved, so it is unclear how the capped RNA fragment interacts with the vRNA template on a structural level [43].

## Cap Snatching in the Host Cell

Influenza RdRP can utilise a wide variety of capped RNA transcripts in the host cell for cap snatching. In this section we discuss which host RNAs are targets for cap snatching, and recent insights into how cap snatching impacts on host transcription by Pol II.

### Targets of Cap Snatching

Just as structural techniques have given insight into the molecular mechanisms of cap snatching, several recent RNA sequencing-based studies have built a picture of which nascent Pol II transcripts are targeted by influenza virus 47, 48, 49, 50. As well as mRNAs, influenza RdRP has been shown to target a wide variety of Pol II-transcribed noncoding RNAs, including small nucleolar RNAs (**snoRNAs**) and small nuclear RNAs (**snRNAs**) 48, 49.

There is some debate over whether specific host transcripts are targeted, or if cap snatching is opportunistic and influenza RdRP simply cap snatches the most abundant nascent capped RNAs. Studies have aimed to examine the cap snatching repertoire by sequencing the 5′ end of viral mRNAs and mapping the 10–13 nt originating from capped RNA fragments back to the donor host transcripts. These studies show that sequences originating from host snoRNAs and snRNAs are very abundant in viral mRNAs 48, 49. U1 and U2, noncoding RNA components of the spliceosome, are the most frequent targets of cap snatching at 3.3% and 3.5% of all viral transcripts, respectively [49]. However, comparing the frequency of snoRNA and snRNA cap snatching with the relative abundance of these RNAs in the host cell suggests that their high cap-snatching frequency is a result of their abundance [50].

Interestingly, multiple studies have observed heterogeneity in cap snatching between different influenza virus genome segments. Specifically, these studies show that different genome segments use different lengths of capped RNA fragments 47, 48. In influenza A/Hong Kong/1/68 (H3N2), the viral genome segments encoding PB1 and PB2 were shown to prefer shorter capped RNA fragments of 11 nt, compared to 12 nt for the other genome segments [47]. In a different study on influenza A/WSN/33 (H1N1), PA and M genome segments were also shown to prefer the shorter 11 nt primers [48]. The preference for shorter capped RNA fragments has been linked to a U/C polymorphism at position 4 on the 3′ end of the vRNA template [47].

Using the same sequencing approach, Koppstein *et al*. observed that up to 30% of influenza mRNAs contain short insertions at the 5′ end, often GCG or GCA trinucleotides depending on the vRNA template [48]. The authors suggest a ‘prime-and-realign’ mechanism acting during viral transcription initiation, in which the vRNA template shifts backwards and reanneals to the capped RNA primer [48]. Recent *in vitro* data have provided mechanistic insight into this process, and suggest that the function of prime and realign is to enable transcription initiation using suboptimal capped RNA primers – for example, capped RNAs which are shorter than normal or which base pair weakly with the vRNA template [51].

### The Impact of Cap Snatching on the Host Cell

**Figure 4:**
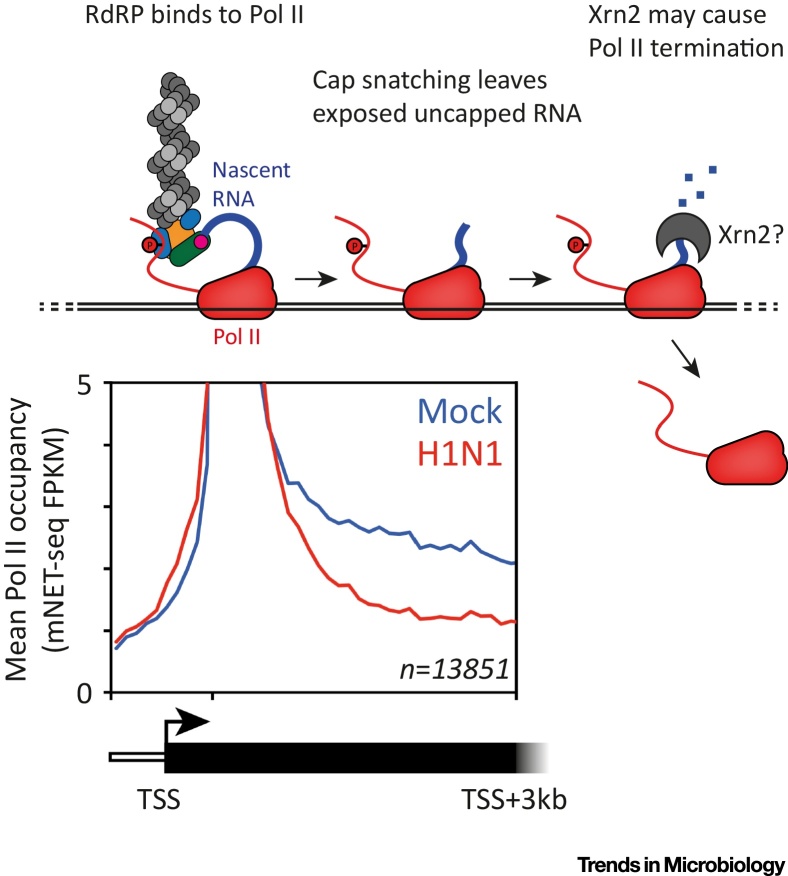
Pol II Termination as a Result of Cap Snatching. Sequencing data (bottom) show that Pol II occupancy, measured in mNET-Seq ‘fragments per kilobase per million mapped reads’, is decreased in gene bodies during influenza virus infection (reproduced from Bauer *et al*. (2018) [32] under the Creative Commons Attribution License (CC BY)). This indicates that influenza virus infection induces premature Pol II termination. In the proposed model (top), cap snatching by influenza RdRP leaves uncapped RNA with an exposed 5′ monophosphate emerging from the Pol II active site. This RNA is a substrate for Xrn2, a host exoribonuclease, which induces Pol II termination according to the torpedo model. TSS indicates transciption start site.

Influenza virus uses a variety of methods to suppress host antiviral responses, including inhibition of Pol II transcription [52]. Recently, Bauer *et al*. have shown that influenza virus infection causes a dramatic loss of Pol II occupancy on host genes on a genome-wide basis [32]. This was demonstrated by sequencing nascent host RNAs bound to Pol II, in influenza virus-infected cells, in a procedure called mNET-Seq (mammalian nascent elongating transcript sequencing). From these data the authors suggest that cap snatching by influenza RdRP results in termination or inhibition of Pol II transcription [32]. One current model for this mechanism is that decapped RNA on Pol II can be degraded by host Xrn2, which leads to Pol II termination by the torpedo model (Figure 4) 32, 52. Inducing Pol II termination may help to avoid the induction of an antiviral response in the host cell, as this is dependent on successful interferon expression, which is dependent on transcription of protein-coding mRNAs by Pol II [53]. It has been estimated that 8 h postinfection with influenza virus, over half of all mRNA in a cell is viral mRNA [54]. This is a striking shift which may be contributed to by the host shut-off function of cap snatching.

## Concluding Remarks

Recent studies have provided insights into molecular details of the cap snatching process and how this links to transcription initiation by influenza RdRP. Biochemical and structural studies have revealed that influenza RdRP in a vRNP is targeted to Pol II using Ser5P CTD binding sites, which vary between influenza types. While these interactions are clearly important, it is unlikely that Pol II CTD binding alone accounts for all the interplay between influenza RdRP and Pol II in cap snatching (see Outstanding Questions). Pol II Ser5P CTD binding to influenza RdRP promotes a transcriptionally competent conformation which enhances nascent capped RNA cleavage and transcription initiation [27]. This provides insight into how influenza RdRP conformation is regulated during different stages of the viral life cycle. Pol II CTD interactions may also contribute to the downregulation of viral transcription in the later stages of viral infection, as newly synthesised RdRP could compete with vRNPs for binding to cellular Pol II [26].

Nascent Pol II-transcribed RNAs appear to be targeted based on abundance, and heterogeneity in cap snatching between different viral genome segments has been observed 47, 48, 50. Influenza RdRP is able to utilise a prime-and-realign mechanism to support productive elongation from suboptimal capped RNA fragment primers 48, 51. Cap snatching has also been implicated in host shut-off [32]. The importance of cap snatching has resulted in it attracting attention as a drug target, and promising novel antivirals are emerging which target the influenza RdRP endonuclease and m^7^G cap-binding activities 8, 55, 56. There are other potential therapeutic targets in cap snatching, such as the newly identified Pol II CTD binding sites on influenza RdRP, and it is likely that more targets will emerge as further details of the cap-snatching process come to light.

Outstanding Questions What is the structure of the influenza vRNP–Pol II complex? Do host proteins aid this interaction during cap snatching?

When, in viral transcription, do influenza vRNPs dissociate from the Pol II CTD?

Is heterogeneity in cap snatching between viral genome segments important for influenza virus?

How does cap snatching lead to Pol II termination? How important is cap snatching for host shut-off by influenza virus?

## Glossary

**CDK7**: cyclin-dependent kinase 7. A kinase with functions in cell cycle control and Pol II transcription. It phosphorylates Ser5 on the Pol II CTD.
**CDK9**: cyclin-dependent kinase 9. A kinase involved in Pol II transcription which complexes with a cyclin to form the P-TEFb complex. It phosphorylates Ser2 on the Pol II CTD.
**HTat-SF1**: HIV Tat-specific factor 1. A transcription factor which is involved in the elongation phase of Pol II transcription.
**P-TEFb**: positive transcription elongation factor b. The name for a complex of CDK9 and Cyclin-T, which is involved in the elongation phase of Pol II transcription.
**RNA exosome complex**: a multisubunit complex with exoribonuclease activity. It is involved in RNA regulation.
**RPB1**: the largest subunit of RNA polymerase II. It has an unstructured C terminal domain consisting of 52 repeats of 7 amino acids.
**snoRNA**: small nucleolar RNA. A class of noncoding RNAs that are involved in chemical modification of other RNAs. They are transcribed by RNA polymerase II or III.
**snRNA**: small nuclear RNA. A class of non-coding RNAs that include RNA components of the spliceosome. They are transcribed by RNA polymerase II.
**SPT5**: transcription elongation factor SPT5. It forms the DRB sensitivity-inducing factor complex by binding to SPT4. This complex promotes Pol II promoter-proximal pausing and associates with mRNA capping enzymes.
**TFIIH**: transcription Factor II H. A multisubunit complex involved in the initiation of Pol II transcription.
